# HMGB1-TLR4 signaling-mediated neuroinflammation contributes to the pathogenesis of infantile epileptic spasms syndrome in rats

**DOI:** 10.3389/fneur.2026.1848952

**Published:** 2026-06-26

**Authors:** Hui Chen, Jianmin Zhong, Yong Chen, Huaping Wu, Ruiyan Wang, Zhaoshi Yi, Xingying Zeng

**Affiliations:** 1 Department of Neurology, Children's Hospital of Jiangxi Province, Nanchang, China; 2 Central Laboratory, Children's Hospital of Jiangxi Province, Nanchang, China

**Keywords:** adrenocorticotropic hormone, high mobility group box 1, infantile epileptic spasms syndrome, neuroinflammation, Toll-like receptor 4

## Abstract

**Background:**

Infantile epileptic spasm syndrome (IESS) is a severe age-dependent epileptic encephalopathy in infancy with poor prognosis and unclear pathogenesis. Neuroinflammation plays a pivotal role in epileptogenesis, and the high-mobility group box 1 protein (HMGB1)-Toll-like receptor 4 (TLR4) axis acts as a core mediator of neuroinflammation. However, its specific role in IESS remains elusive.

**Objective:**

This study aimed to explore the HMGB1-TLR4-mediated neuroinflammatory mechanism in a rat model of IESS induced by prenatal stress combined with NMDA, and to evaluate the effects of anti-HMGB1 neutralizing antibody and adrenocorticotropic hormone (ACTH) on epileptic seizures and neuroinflammation, so as to provide novel therapeutic targets for clinical practice.

**Methods:**

Pregnant Sprague-Dawley rats were randomly divided into prenatal stress (PS) and non-prenatal stress (NPS) groups. PS rats received cold water immersion and hot air drying, while NPS rats were reared normally. On postnatal day 12 (P12), offspring in the PS group were intraperitoneally injected with NMDA to establish the IESS model, and the NPS group was assigned to blank control (BC) and negative control (NC) subgroups. Model rats were randomly divided into ACTH, anti-HMGB1, ACTH+anti-HMGB1, normal saline, and untreated groups. After intervention on P13, NMDA was re-administered, and seizure latency and severity score were recorded. At the end of the experiment, the expression of HMGB1 and TLR4 in brain tissue was detected, HMGB1 co-localization was observed, and the levels of iNOS, Arg1 and cytokines (IL-1β, IL-2R, IL-8, TNF-α) were measured.

**Results:**

Prenatal stress combined with NMDA successfully established a stable IESS model in young rats. The expression of HMGB1, TLR4, iNOS, IL-1β, IL-2R, IL-8 and TNF-α was significantly upregulated, while Arg1 was markedly downregulated. Treatment with ACTH, anti-HMGB1, and their combination prolonged seizure latency, reduced seizure severity, downregulated HMGB1 and TLR4 expression, suppressed HMGB1 levels in neurons, astrocytes and activated microglia, inhibited iNOS and proinflammatory cytokines, and promoted Arg1 expression, with the combined intervention showing the optimal efficacy.

**Conclusion:**

Prenatal stress combined with NMDA activates the HMGB1/TLR4 pathway and neuroinflammation in IESS rats. ACTH and anti-HMGB1, alone or in combination, alleviate neuroinflammation by inhibiting this pathway to ameliorate IESS, and the combined therapy yields the best therapeutic effect.

## 1 Introduction

Infantile Epileptic Spasms Syndrome (IESS) is a severe form of epilepsy predominantly affecting infants and young children, with an onset age typically between 1 and 24 months and a peak incidence at 3 to 12 months of age (1). The main clinical features are epileptic spasms, including flexor spasms, mixed (flexor/extensor) spasms and extensor spasms (2). The estimated incidence of IESS is 30 per 100,000 live births; some studies have suggested a higher incidence in high-latitude regions (Sweden, Finland, Denmark) (3–6). The etiology of IESS is complex and diverse, mainly including structural, genetic, infectious and metabolic factors, and the cause remains unknown in some cases even after relevant examinations. Although the clinical characteristics and an increasing number of etiologies of IESS have been identified, the specific pathogenesis of IESS remains unclear, which hinders the development of therapeutic regimens (7–11). The unclear pathogenesis poses great challenges to the treatment of IESS; some children with IESS still suffer from recurrent epileptic spasms despite active treatment, which can lead to severe cognitive impairment and developmental delay, imposing a huge burden on families and society (12–14). Therefore, further research on the pathogenesis of IESS is crucial for its diagnosis and treatment, improving the prognosis of children with IESS and reducing the burden on families and society.

In recent years, numerous studies have found a close association between epilepsy and neuroinflammation. Sustained activation of neuroinflammation can lead to recurrent epileptic seizures, and neuroinflammation is a promising target for therapeutic intervention (15, 16). Current first-line therapeutic drugs for IESS include adrenocorticotropic hormone (ACTH), prednisolone and vigabatrin (17, 18). Among them, hormonal therapy (ACTH, prednisolone) is the first-line preferred regimen only for IESS, but not for other types of epilepsy, suggesting the particularity of IESS as an epileptic syndrome. Considering the immunomodulatory and anti-inflammatory effects of hormones, neuroimmunity and inflammation may be involved in the pathogenesis of IESS. A multicenter prospective trial in southern China investigated the short-term efficacy and immune mechanism of ACTH in the treatment of infantile spasms, and found that approximately 30% of children with IESS had a short-term response to ACTH treatment, and the proportion of CD3+CD8+ T cells and the CD3+CD4+/CD3+CD8+ ratio may have predictive value for the efficacy of ACTH (19). Another study found that patients with IESS exhibited reduced CD4+ T cells, altered CD4/CD8 ratio and decreased production of tumor necrosis factor-α (TNF-α) in CD4+ T cells. The elevated level of activated CD8+ T cells in patients with IESS was significantly correlated with clinical severity (20). A previous study (21) found that serum levels of IL-2, TNF and IFN in children with IESS were higher than those in the normal control group, suggesting that cytokine imbalance may contribute to the pathogenesis of IESS. Our team has long been committed to clinical research on prednisone in the treatment of IESS, and previous studies have shown that oral prednisone is effective and safe for IESS (22, 23). In addition, our previous research also found that serum levels of IL-2R, IL-8 and TNF-α in the IESS group were higher than those in the healthy control group before oral prednisone treatment, and these levels decreased significantly after treatment with the improvement of clinical symptoms in children with IESS, indicating the presence of neuroinflammation or immune dysfunction in children with IESS, and the therapeutic effect of prednisone on IESS may be related to its ability to regulate and improve immune dysfunction in these children (24). In summary, the above studies indicate that neuroimmunity and inflammation are involved in the pathogenesis of IESS. However, due to the complexity of the neuroinflammatory network, it is still unclear how neuroinflammatory dysregulation leads to IESS, and its key signaling pathway remains undefined. Identifying an objective and key signaling pathway is crucial, as it not only provides a theoretical basis for the pathogenesis of IESS but also facilitates its clinical diagnosis and treatment.

High mobility group box 1 (HMGB1) has attracted increasing attention in recent years. It is a highly conserved and widely distributed non-histone DNA-binding protein involved in a variety of cellular physiological functions, such as maintaining the stability of nucleosome structure, regulating gene transcription, and mediating DNA replication and repair (25, 26). With in-depth research, HMGB1 has been identified as an important cellular inflammatory factor and a key protein factor in the inflammatory network. When the body is stimulated by various factors, HMGB1 can be actively released by activated immune cells or passively released into the extracellular environment by necrotic or damaged cells. Acetylation of HMGB1 is required during its translocation; after being transported to the extracellular space, it is modified under the action of reactive oxygen species to form a stable HMGB1 disulfide form, which then binds to its receptors and triggers inflammatory responses by activating signaling pathways such as nuclear factor-κB (NF-κB), leading to tissue damage (26, 27). HMGB1 has a variety of receptors, among which Toll-like receptor 4 (TLR4), Toll-like receptor 2 (TLR2) and receptor for advanced glycation end products (RAGE) are the most important. Notably, the inflammatory signaling pathway mediated by the binding of HMGB1 to TLR4 is closely associated with epilepsy. As one of the innate immune receptors, TLR4 is widely distributed in the nervous system, including neurons, microglia and astrocytes, and is involved in a variety of bacterial infections and endogenous injuries, serving as an important receptor mediating macrophage activation, cytokine production and tissue damage (28). A large number of previous studies have shown a close relationship between the HMGB1/TLR4 signaling pathway and the occurrence of epilepsy. A previous study found that the expression levels of HMGB1, TLR4, TNF-α and IL-1β in the hippocampal tissues of a rat model of mesial temporal lobe epilepsy and children with mesial temporal lobe epilepsy after surgery were significantly higher than those in the normal control group, indicating that HMGB1-TLR4 signaling pathway-mediated neuroinflammation is involved in the occurrence of mesial temporal lobe epilepsy seizures (29). A recent study found that montelukast improved pentylenetetrazol-induced epileptic seizures by regulating the HMGB1/TLR4 pathway, alleviating neuroinflammation, oxidative stress and seizure severity, further confirming that HMGB1/TLR4-mediated neuroinflammation is involved in epileptic seizures (30). Multiple clinical studies have further supported a strong correlation between epilepsy and the HMGB1/TLR4 signaling pathway (31–33). As a specific epileptic syndrome (the only epileptic syndrome for which hormones are the first-line therapeutic drug, with characteristic epileptic spasm manifestations), IESS is different from other types of epilepsy and should have its unique pathogenesis requiring independent research. Unfortunately, evidence on the correlation between the HMGB1/TLR4 signaling pathway and IESS is extremely rare, only briefly mentioned in a previous clinical study. This study included 180 children with new-onset epilepsy (67 cases of generalized tonic-clonic seizures, 92 cases of focal motor seizures, 21 cases of IESS) and 40 healthy children, and the results showed that serum HMGB1 and IL-1β concentrations in the epilepsy group within 24 h after seizure were significantly higher than those in the control group, with a particularly significant increase in children with IESS (34). In summary, it is hypothesized that the HMGB1-TLR4 signaling pathway-mediated neuroinflammatory mechanism may be one of the pathogenesis of IESS, but further experiments are needed to confirm this hypothesis.

In this study, an animal model of IESS was established to detect the mRNA and protein expressions of HMGB1 and TLR4 in the brain tissues of IESS rats, as well as the expression levels of inflammatory factors (IL-1β, IL-2R, IL-8, TNF-α) that have been reported to be elevated in previous clinical studies by our research team and other relevant clinical investigations. This was performed to elucidate the role of HMGB1-TLR4 signaling pathway-mediated neuroinflammation in the pathogenesis of IESS. Additionally, anti-HMGB1 antibody and ACTH, a classic therapeutic drug for IESS, were used to intervene in IESS rats to observe their effects on epileptic seizures and neuroinflammation, thereby providing novel therapeutic targets for the clinical management of IESS.

## 2 Materials and Methods

### 2.1 Animals

Ten female SD rats (3 months old) were purchased from Beijing SPF Biotechnology Co., Ltd. (License No.: SCXK (Beijing) 2024-0001) and raised under the conditions of 20~26 °C, 40–70% humidity, and a 12 h/12 h light/dark cycle. Female SD rats were caged with male SD rats, and vaginal plugs were checked daily. The presence of a vaginal plug was considered a sign of pregnancy. Pregnant rats were randomly divided into a prenatal stress exposure group and a non-prenatal stress exposure group. The prenatal stress exposure group received stress exposure from gestational day 1. Pups were nursed by their mothers, weighed and recorded one by one on postnatal day 12, and those with significant weight differences were excluded. Pups born to pregnant rats without prenatal stress exposure were defined as the non-model group and randomly divided into a blank control (BC) group and a negative control (NC) group; pups born to pregnant rats with prenatal stress exposure were defined as the model group. The rat model of IESS was established by prenatal stress combined with intraperitoneal injection of N-Methyl-D-aspartic acid (NMDA), referring to previous studies (35, 36). After successful induction of IESS, the model group was further randomly divided into 5 subgroups: Model Group I, Model Group II, Model Group III, Model Group IV and Model Group V. All animal experiments, feeding and management in this study were strictly in accordance with relevant regulations and requirements, and approved by the Experimental Animal Ethics Committee.

The establishment method of the rat model of IESS was as follows: (1) Prenatal stress exposure: Pregnant rats were randomly divided into two groups: a prenatal stress (PS) group and a non-prenatal stress (NPS) group. Pregnant rats in the PS group were forced to soak in cold water (4 °C) in an organic glass cylinder (50 cm in height, 20 cm in diameter) with a water depth of approximately 35 cm to prevent the rats from standing without touching the bottom of the cylinder with their tails. After 5 min of immersion, the rats were removed, dried in a heated container for 10 min, and then returned to their cages. Stress was applied daily at 17:00 from gestational day 1 to delivery. (2) Induction of epileptic seizures (spasms): The day of birth was recorded as P0. At 10:00 on postnatal day 12 (P12), rats in the model group were intraperitoneally injected with NMDA (7.5 mg/kg, prepared to a concentration of 1 mg/mL with 0.9% sodium chloride injection) to induce epileptic seizures; the negative control group was intraperitoneally injected with an equal volume of normal saline, and the blank group received no treatment. The rats were observed for 3 h. Rats with a successfully established IESS model typically exhibited flexor spasms with curled body, flexion of the head, spine, hip and knee joints, and recurrent clustered seizures lasting 3–5 s each. Rats that died, had no seizures, or had seizures without flexor spasms were excluded as model establishment failures.

After successful establishment of the model by prenatal stress combined with intraperitoneal injection of NMDA, interventions were performed at 10:00 on postnatal day 13 (P13): Model Group I (IESS+ACTH): intraperitoneal injection of ACTH (100 IU/kg, prepared to a concentration of 20 U/mL with normal saline, P13, 10:00) (37); Model Group II (IESS+anti-HMGB1): intraperitoneal injection of HMGB1 neutralizing antibody (300 μg/kg) (38); Model Group III (IESS+ACTH+anti-HMGB1): intraperitoneal injection of ACTH (100 IU/kg) and HMGB1 neutralizing antibody (300 μg/kg); Model Group IV (IESS+NS): intraperitoneal injection of an equal volume of normal saline; Model Group V: positive control group, no treatment.

Reinduction of epileptic seizures (spasms): Rats in the model group were intraperitoneally injected with NMDA (12 mg/kg) at 30 min after drug treatment on P13; the blank group received no treatment, and the negative control group underwent the same operations during model establishment and drug treatment, but the drugs were replaced with normal saline.

The seizure latency (time from intraperitoneal injection of NMDA to the onset of epileptic seizures) and seizure severity score were recorded after intraperitoneal injection of NMDA on P12 and P13, respectively. The scoring criteria were as follows: 0 (no abnormal response), 1 (quiet wheezing), 2 (increased activity, irritability, tail flicking), 3 (self-biting or mutual biting), 4 (number of curling-up spasm: n ≤ 4), 5 (number of curling-up spasm: 5 ≤ n ≤ 14), 6 (number of curling-up spasm: 15 ≤ n ≤ 29), 7 (number of curling-up spasm: n ≥ 30), 8 (involuntary choreiform movements of limbs after tonic-clonic seizures), 9 (death) (37).

Rats were euthanized at 24 h after NMDA injection on P13 (i.e., P14) to collect relevant samples. The euthanasia method was performed as follows: Rats were anesthetized by intramuscular injection of a combination of Zoletil^®^ 50 (VIRBAC, batch number: BN9UAKA) at a dose of 25 mg/kg and Sumianxin II Injection (also known as Xylazine Hydrochloride Injection, Shengda Animal Pharmaceutical Co., Ltd., Dunhua City, Jilin Province, China; batch number: 20240607) at a dose of 12.5 mg/kg. Subsequent to successful anesthesia, euthanasia was achieved by excessive blood collection from the cardiac region until cardiac arrest.

### 2.2 qPCR Detection

Total RNA was extracted from the brain tissue of one side (the same side in each group) using Trizon reagent (CW0580S, CWBIO), and mRNA was extracted using an RNA ultra-pure extraction kit (CW0581M, CWBIO). The concentration and purity (OD260/OD280) of mRNA were determined using an ultraviolet-visible spectrophotometer (NP80, NanoPhotometer). cDNA was synthesized using an RNA reverse transcription kit (R223-01, Vazyme), and quantitative real-time PCR was performed using a fluorescent PCR instrument (CFX Connect™, Bio-Rad Laboratories (Shanghai) Co., Ltd.). The reaction procedure was as follows: pre-denaturation at 95 °C for 10 min; denaturation at 95 °C for 10 s; annealing at 58 °C for 30 s; extension at 72 °C for 30 s, for 40 cycles. The threshold cycle (Ct) was individually set for each target gene and GAPDH. The fixed 40 amplification cycles was our experimental setup, and the Ct value was determined based on the logarithmic amplification phase rather than the fixed terminal cycle. GAPDH was used as the internal reference, and the relative expression level of genes was calculated according to the 2^−ΔΔ*Ct*^ method (39). ΔCt = Ct (target gene)–Ct (GAPDH). ΔΔ*Ct*= ΔCt (model/experimental group)–ΔCt (control group). The relative mRNA expression fold change was calculated as: Relative expression = 2^−ΔΔ*Ct*^. All statistical analyses were performed based on the normalized relative expression values derived from the 2^−ΔΔ*Ct*^ calculation. The primer sequences are shown in the Table 1 below.

**Table 1:** Primer sequences used for qPCR.

Primer name	Primer sequences (5′-3′)
GAPDH F	GACAACTTTGGCATCGTGGA
GAPDH R	ATGCAGGGATGATGTTCTGG
HMGB1 F	TGACAAGGCTCGTTATGAAAG
HMGB1 R	TTCTTCGCAACATCACCAAT
TLR4 F	CCAGAGCCGTTGGTGTATCT
TLR4 R	CCAGAGCCGTTGGTGTATCT

### 2.3 Western Blot (WB) detection

Brain tissue from one side (the same side in each group) was collected, added with RIPA lysis buffer (C1053, Beijing Pulilai Gene Technology Co., Ltd.), and ground using a tissue grinder to extract total tissue protein. The mixture was centrifuged at 12,000 r/min at 4 °C for 10 min using a high-speed centrifuge (5424R, Eppendorf), and the supernatant was collected. The total protein was quantified using a BCA protein quantification kit. After denaturation of protein samples, sodium dodecyl sulfate-polyacrylamide gel electrophoresis (SDS-PAGE) was performed for 1.5 h, followed by electrotransfer to a membrane at a constant current of 300 mA for 1 h. The PVDF membrane (Millipore) was blocked with skimmed milk powder, incubated with primary antibodies at 4 °C overnight, and then incubated with secondary antibodies at room temperature for 2 h the next day. The PVDF membrane was soaked with luminescent solution and developed in an ultra-sensitive chemiluminescence imaging system (Tanon-5,200, Shanghai Tanon Science & Technology Co., Ltd.). The antibodies used and their corresponding dilution ratios are shown in the Table 2 below. The gray value of each protein band was quantified by ImageJ software. The target protein gray value was normalized to the internal reference protein gray value to obtain the relative protein expression level.

**Table 2:** Antibody information.

Antibody name	Dilution
Mouse Anti-GAPDH (HC301, TransGen Biotech)	1/2000
HRP conjugated Goat Anti-Mouse IgG (H+L)(GB23301, Servicebio)	1/2000
Rabbit Anti TLR4(A5258, abclonal)	1/1000
Rabbit Anti HMGB1(A2553, abclonal)	1/1000
HRP conjugated Goat Anti-Rabbit IgG (H+L)(GB23303, Servicebio)	1/2000

### 2.4 Detection of brain tissue cytokines by enzyme-linked immunosorbent assay (ELISA)

Brain tissue from one side (the same side in each group) stored at−80 °C was thawed at room temperature and rinsed with normal saline for 1–2 min. The water on the brain tissue was blotted dry with filter paper and weighed using an electronic balance. The brain tissue was transferred to a 10 mL centrifuge tube, and normal saline was added at a ratio of 100 mg brain tissue: 900 μl normal saline. The brain tissue was ground using a high-speed tissue grinder to prepare brain tissue homogenate, which was centrifuged at 4,000 rpm/min at 4 °C for 15 min. The supernatant was collected, aliquoted into EP tubes, and stored at−20 °C for later use. The levels of brain tissue cytokines IL-1β, IL-2R, IL-8 and TNF-α were detected by ELISA using rat IL-1β ELISA kit (MM-0047R1, MeiMian), rat IL-2R ELISA kit (MM-0011R1, MeiMian), rat IL-8 ELISA kit (MM-0175R1, MeiMian), and rat TNF-α ELISA kit (MM-0180R1, MeiMian), respectively. The experimental procedures were as follows: (1) Take out the required strip wells from the aluminum foil bag after equilibrating at room temperature for 20 min, and seal the remaining strip wells in a self-sealing bag and store at 4 °C. (2) Set up standard wells and sample wells, and add 50 μl of standard substances with different concentrations to each standard well; (3) Add 50 μl of the sample to be tested to each sample well; no addition to blank wells; (4) Except for blank wells, add 100 μl of horseradish peroxidase (HRP)-labeled detection antibody to each standard well and sample well, seal the reaction wells with a plate sealer, and incubate in a 37 °C water bath or incubator for 60 min. (5) Discard the liquid, pat dry on absorbent paper, fill each well with washing solution (350 μl), let stand for 1 min, discard the washing solution, pat dry on absorbent paper, and repeat plate washing 5 times; (6) Add 50 μl of substrate A and substrate B to each well, and incubate at 37 °C in the dark for 15 min; (7) Add 50 μl of stop solution to each well, and determine the OD value of each well at a wavelength of 450 nm within 15 min. (8) Plot a standard curve with the measured OD value of the standard substance as the abscissa and the concentration value of the standard substance as the ordinate, and calculate the concentration of the sample according to the OD value of the sample.

### 2.5 Immunohistochemical detection

Rat brain tissue sections were baked, deparaffinized, hydrated, and subjected to antigen retrieval with citrate buffer. After blocking with 5% BSA, the sections were incubated with primary antibodies against rabbit anti-iNOS (Affinity, AF0199, 1/100) and rabbit anti-Arg1 (Affinity, DF6657, 1/100) at 4 °C overnight, followed by incubation with HRP-labeled goat anti-rabbit IgG (H+L) (ZB-2,301, Zhongshan Jinqiao, 1/100). The sections were developed with DAB, counterstained with hematoxylin and blued, dehydrated, cleared, mounted, and observed under a microscope (CX43, OLYMPUS). The detected brain region was specified as the hippocampus. For field selection, 3 random non-overlapping visual fields were selected from brain tissue section under high magnification, and optical density was analyzed quantitatively.

### 2.6 Double immunofluorescence staining

Tissue sections were baked, deparaffinized, hydrated, and subjected to antigen retrieval with citrate buffer. The sections were permeabilized with 0.5% Triton X-100 at room temperature for 15 min. After washing with PBS, the sections were blocked with 5% BSA at 37 °C for 30 min. Primary antibody Rabbit Anti-HMGB1 (Abclonal, A19529, 1/200) was added dropwise to the slides and incubated at 4°C overnight; the next day, after the slides were rewarmed to room temperature, they were washed with PBS 3 times for 3 min each, and HRP-labeled goat anti-rabbit IgG (H+L) (ZB-2,301, Zhongshan Jinqiao, 1/200) was added dropwise and incubated at room temperature for 50 min. CY3-TSA (G1223, Servicebio, 1/1,500) was added and incubated at room temperature for 10 min in the dark. After heat retrieval and permeabilization, the slides were washed with PBS 3 times and blocked again with 5% BSA at 37 °C for 30 min. Primary antibodies Rabbit Anti-ED1 (Abclonal, A2905, 1/100), Rabbit Anti-NeuN (Proteintech, 26975-1-AP, 1/200) and Rabbit Anti-GFAP (Afffinity, DF6040, 1/200) were added dropwise and incubated overnight at 4 °C in a wet box. After the slides were rewarmed to room temperature and washed with PBS 3 times, diluted goat anti-rabbit IgG/488 (ZF-0511, Zhongshan Jinqiao, 1/100) was added dropwise and incubated at 37 °C for 45 min in a wet box. The cell nuclei were counterstained with DAPI (KGA1808-50, KeyGen BioTECH) for 4 min, the slides were washed and the liquid was blotted dry, mounted with mounting medium containing anti-fluorescence quencher, and observed under a fluorescence microscope (BX53, OLYMPUS).

### 2.7 Data analysis

Graphpad Prism 9.0 software was used for graph plotting and statistical analysis. One-way analysis of variance (ANOVA) was used for difference analysis, and *P* < 0.05 was considered statistically significant.

## 3 Results

### 3.1 Animal model establishment results

**Figure 1:**
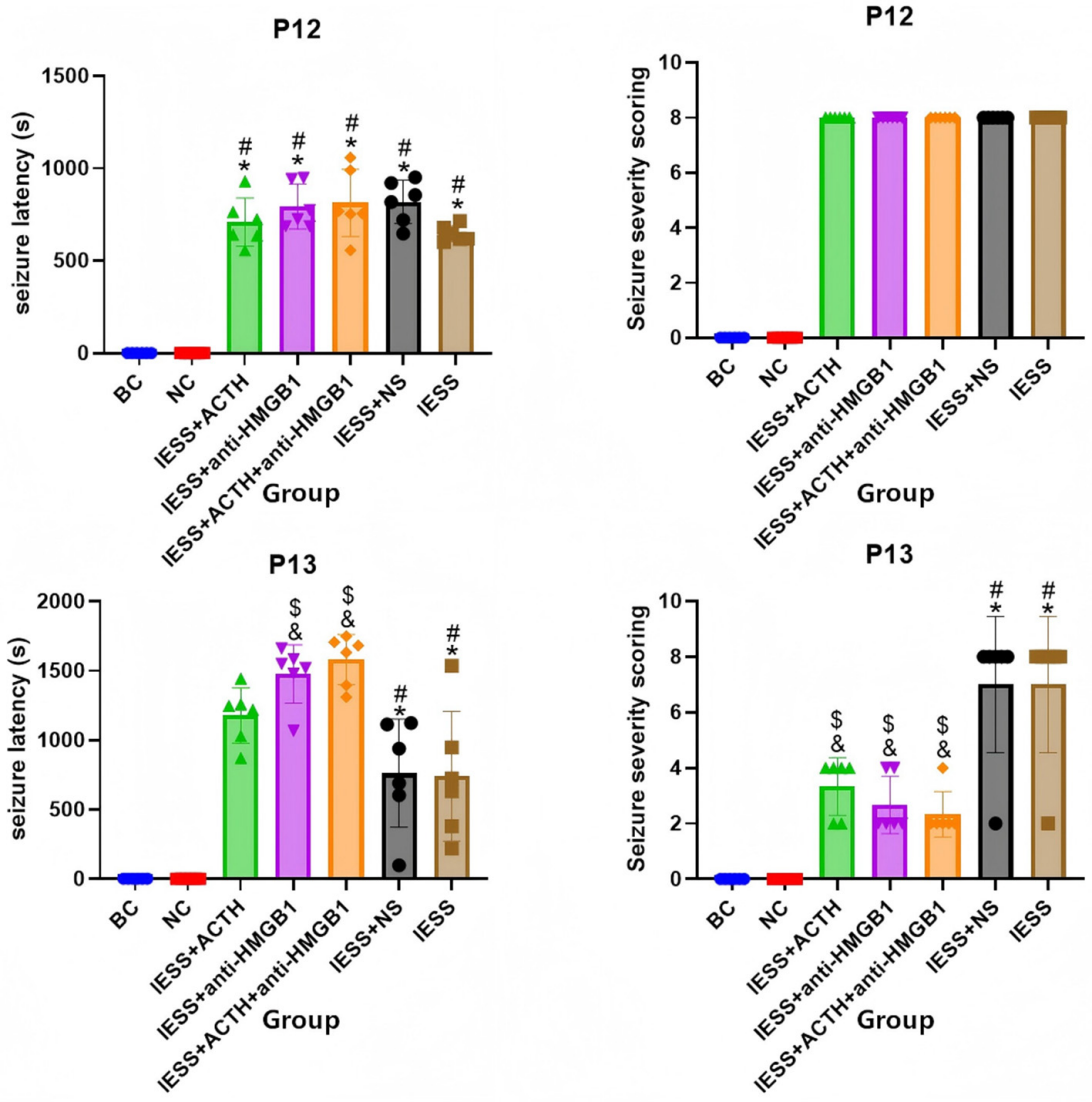
Behavioral results. Experimental grouping: Non-model group (pups from non-prenatal stress pregnant rats) was divided into blank control (BC, no treatment) and negative control (NC, normal saline instead of drugs); Model group (pups from prenatal stress pregnant rats, IESS model induced by prenatal stress + NMDA intraperitoneal injection) was divided into 5 subgroups: Model I (IESS+ACTH, 100 IU/kg intraperitoneal injection), Model II (IESS+anti-HMGB1, 300 μg/kg intraperitoneal injection), Model III (IESS+ACTH+anti-HMGB1), Model IV (IESS+NS, equal volume normal saline), Model V (IESS, positive control, no treatment); Detection indicator: the seizure latency:time from intraperitoneal injection of NMDA to the onset of epileptic seizures; seizure severity score were recorded after intraperitoneal injection of NMDA on P12 and P13, respectively. Statistical significance description: (*P < 0.05 vs. BC group, #*P* < 0.05 vs. NC group, &*P* < 0.05 vs. IESS+NS group, $*P* < 0.05 vs. IESS group) (one-way ANOVA).

The results of seizure latency (Figure 1) showed no significant difference in the seizure latency among all model groups after induction on P12 (*P* > 0.05); the results of seizure severity score (Figure 1) also showed no significant difference among all model groups after induction on P12 (*P* > 0.05). All model rats included in this study exhibited curling-up spasm and involuntary choreiform movements of limbs after tonic-clonic seizures, indicating the successful establishment of the IESS model in young rats. After successful model establishment with NMDA, young model rats were intervened with ACTH, anti-HMGB1 and their combination on P13, and NMDA was intraperitoneally injected again to induce epileptic seizures. The seizure latency and seizure severity score were recorded. The results of seizure latency (Figure 1) showed that ACTH intervention prolonged the seizure latency of IESS young rats with no statistical significance (*P* > 0.05), while anti-HMGB1 and the combination of ACTH and anti-HMGB1 significantly prolonged the seizure latency of IESS young rats. The results of seizure score (Figure 1) showed that ACTH, anti-HMGB1 and their combination significantly reduced the seizure severity score of IESS young rats. These results indicated that ACTH, anti-HMGB1 and their combination could alleviate epileptic seizures in IESS young rats, with the combination intervention showing the best effect.

### 3.2 qPCR Detection results

**Figure 2:**
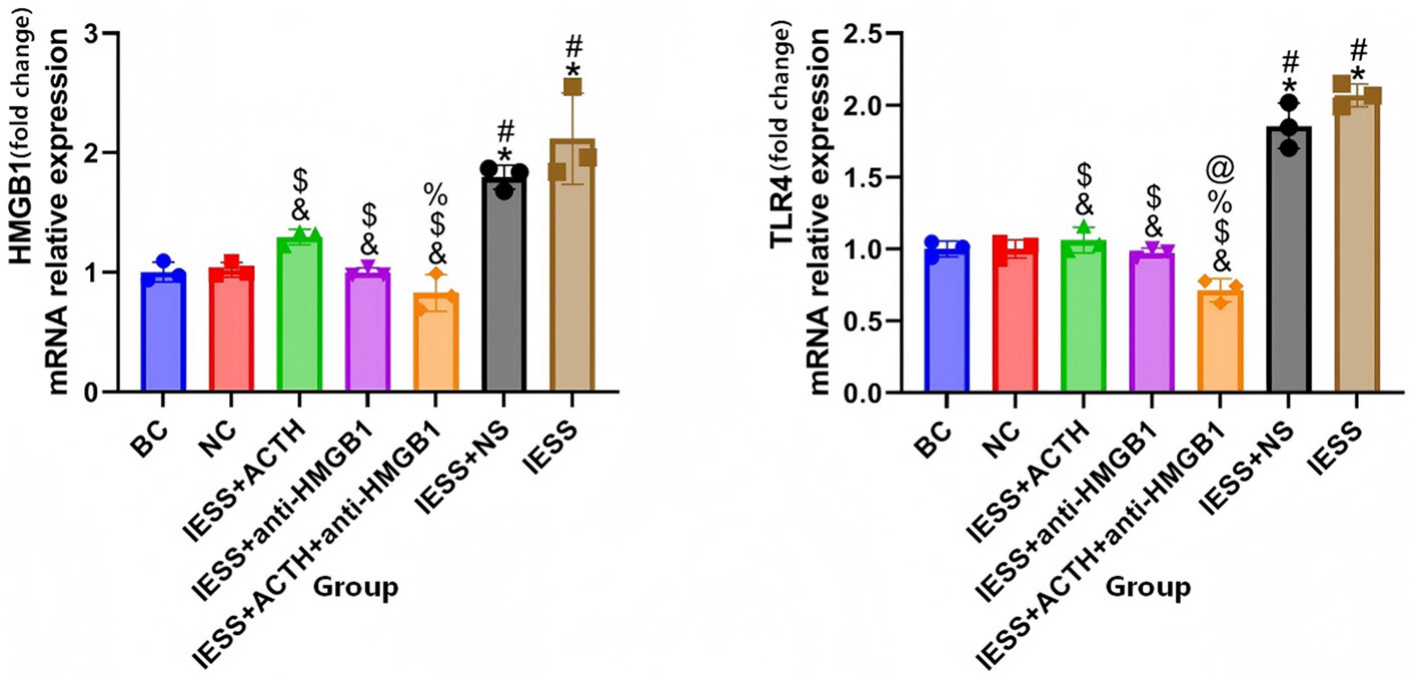
qPCR detection results of brain tissues. Experimental grouping: Non-model group (pups from non-prenatal stress pregnant rats) was divided into blank control (BC, no treatment) and negative control (NC, normal saline instead of drugs); Model group (pups from prenatal stress pregnant rats, IESS model induced by prenatal stress + NMDA intraperitoneal injection) was divided into 5 subgroups: Model I (IESS+ACTH, 100 IU/kg intraperitoneal injection), Model II (IESS+anti-HMGB1, 300 μg/kg intraperitoneal injection), Model III (IESS+ACTH+anti-HMGB1), Model IV (IESS+NS, equal volume normal saline), Model V (IESS, positive control, no treatment); Detection indicator: the mRNA expressions of HMGB1 and TLR4. Detection method: GAPDH was used as the internal reference, and the relative expression level of genes was calculated according to the 2^−ΔΔ*Ct*^ method; Statistical significance description: (*P < 0.05 vs. BC group, #*P* < 0.05 vs. NC group, &*P* < 0.05 vs. IESS+NS group, $*P* < 0.05 vs. IESS group, %*P* < 0.05 vs. IESS+ACTH group, @*P* < 0.05 vs. IESS+anti-HMGB1 group) (one-way ANOVA).

The qPCR detection results (Figure 2) showed that the mRNA expressions of HMGB1 and TLR4 in the IESS+NS group and IESS group were significantly higher than those in the BC group and NC group; compared with the IESS+NS group and IESS group, the mRNA expressions of HMGB1 and TLR4 in the IESS+ACTH group, IESS+anti-HMGB1 group and IESS+ACTH+anti-HMGB1 group were decreased. These results indicated that ACTH, anti-HMGB1 and their combination inhibited the mRNA expressions of HMGB1 and TLR4 in the brain tissues of IESS young rats, with the combination intervention exerting the best effect.

### 3.3 WB Detection results

**Figure 3:**
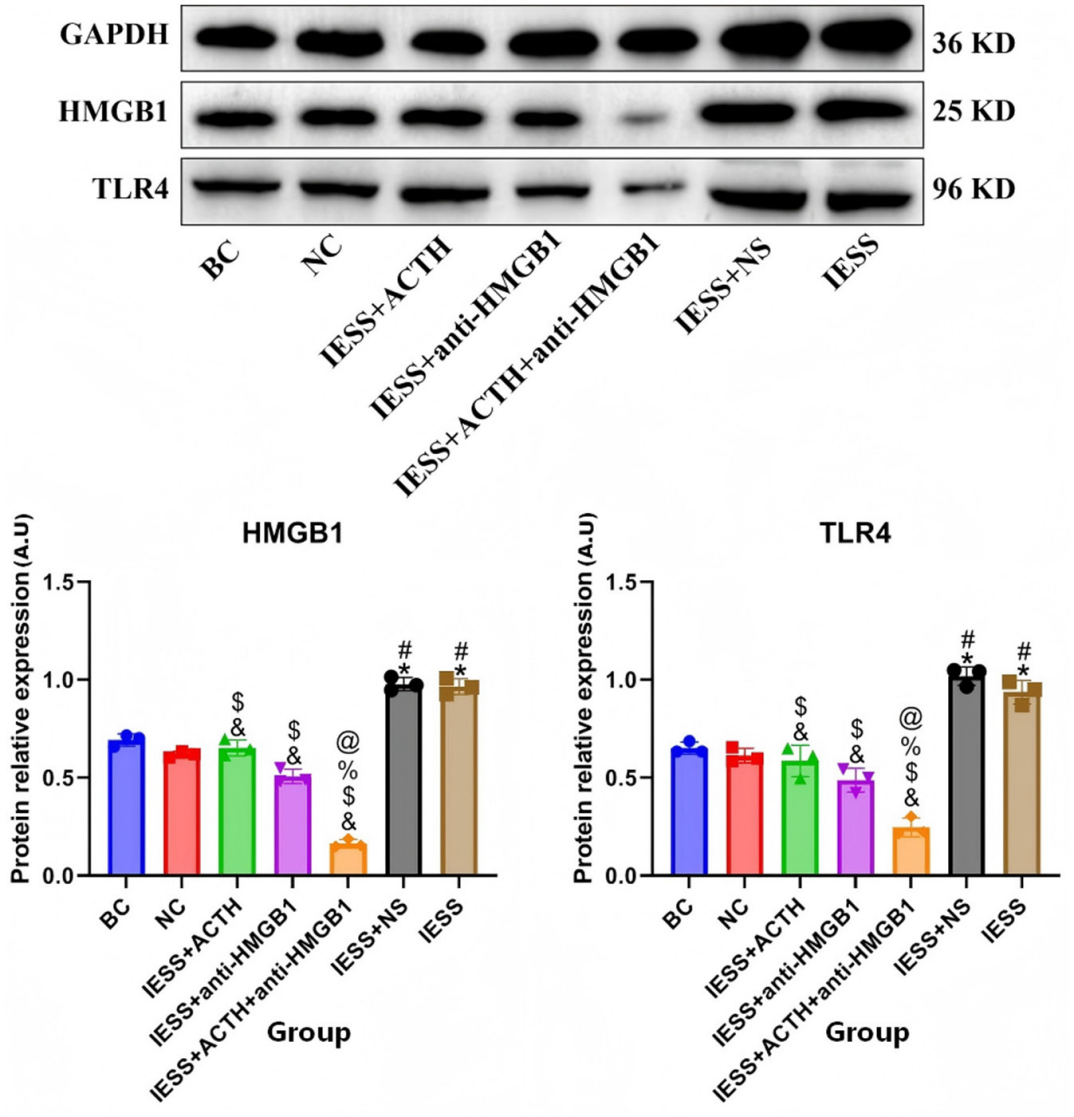
WB detection results of brain tissues. Experimental grouping: Non-model group (pups from non-prenatal stress pregnant rats) was divided into blank control (BC, no treatment) and negative control (NC, normal saline instead of drugs); Model group (pups from prenatal stress pregnant rats, IESS model induced by prenatal stress + NMDA intraperitoneal injection) was divided into 5 subgroups: Model I (IESS+ACTH, 100 IU/kg intraperitoneal injection), Model II (IESS+anti-HMGB1, 300 μg/kg intraperitoneal injection), Model III (IESS+ACTH+anti-HMGB1), Model IV (IESS+NS, equal volume normal saline), Model V (IESS,positive control, no treatment); Detection indicator: the protein relative expressions of HMGB1 and TLR4. Detection method: the protein relative expressions levels in brain tissues detected by Western Blot (WB); Statistical significance description: (^*^P < 0.05 vs. BC group, #*P* < 0.05 vs. NC group, &*P* < 0.05 vs. IESS+NS group, $*P* < 0.05 vs. IESS group, % *P* < 0.05 vs. IESS+ACTH group, @*P* < 0.05 vs. IESS+anti-HMGB1 group) (one-way ANOVA).

The WB detection results (Figure 3) showed that the protein expressions of HMGB1 and TLR4 in the IESS+NS group and IESS group were significantly higher than those in the BC group and NC group; compared with the IESS+NS group and IESS group, the protein expressions of HMGB1 and TLR4 in the IESS+ACTH group, IESS+anti-HMGB1 group and IESS+ACTH+anti-HMGB1 group were significantly decreased. These results indicated that ACTH, anti-HMGB1 and their combination inhibited the protein expressions of HMGB1 and TLR4 in the brain tissues of IESS young rats, with the combination intervention showing the best effect.

### 3.4 ELISA detection results of brain tissue cytokines

**Figure 4:**
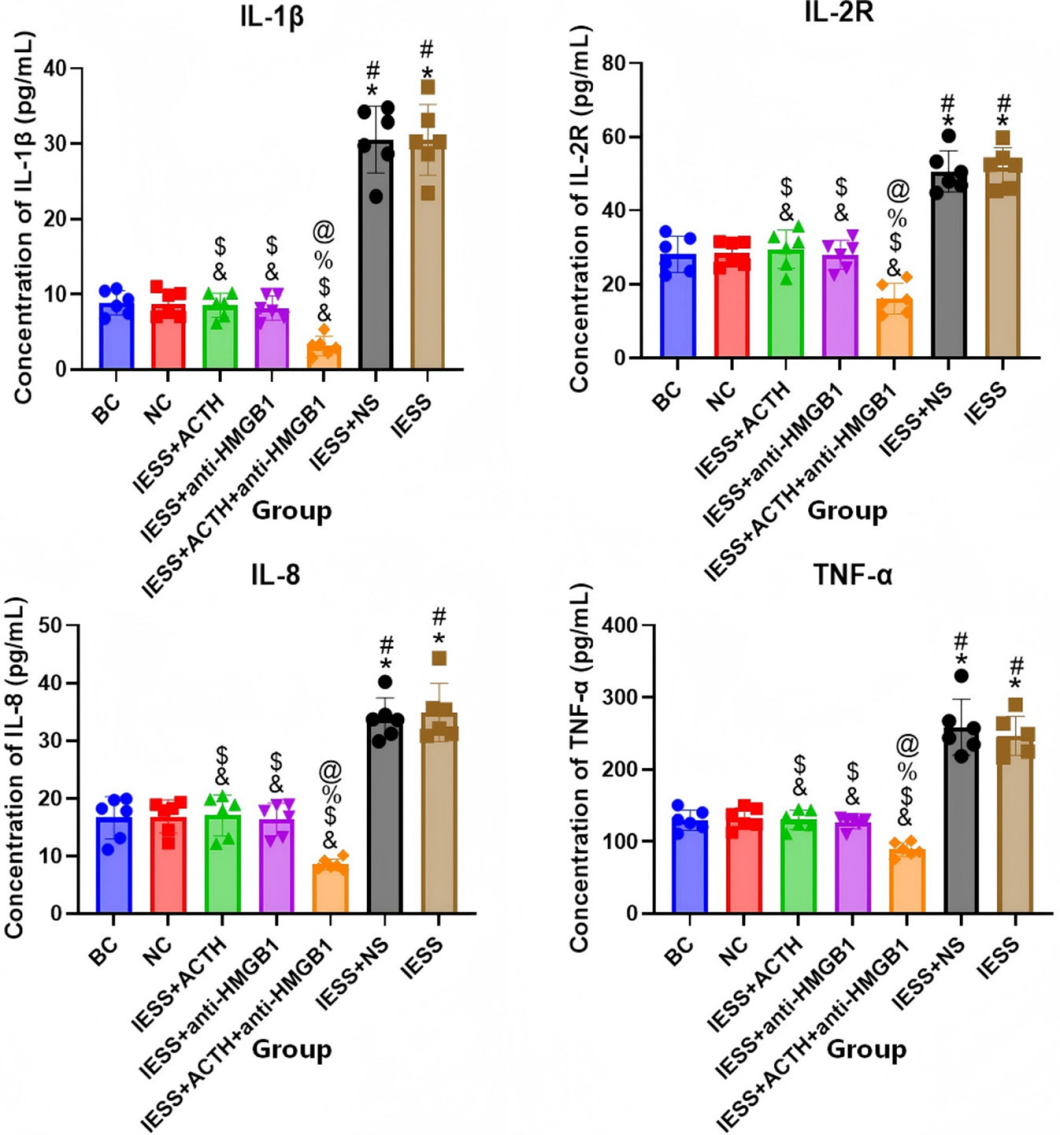
Expression levels of cytokines in brain tissues. Experimental grouping: Non-model group (pups from non-prenatal stress pregnant rats) was divided into blank control (BC, no treatment) and negative control (NC, normal saline instead of drugs); Model group (pups from prenatal stress pregnant rats, IESS model induced by prenatal stress + NMDA intraperitoneal injection) was divided into 5 subgroups: Model I (IESS+ACTH, 100 IU/kg intraperitoneal injection), Model II (IESS+anti-HMGB1, 300 μg/kg intraperitoneal injection), Model III (IESS+ACTH+anti-HMGB1), Model IV (IESS+NS, equal volume normal saline), Model V (IESS, positive control, no treatment); Detection indicator: expression levels of cytokines in brain tissues. Detection method: expression levels of cytokines in brain tissues detected by Enzyme-Linked Immunosorbent Assay (ELISA); Statistical significance description: (*P < 0.05 vs. BC group, #*P* < 0.05 vs. NC group, &*P* < 0.05 vs. IESS+NS group, $*P* < 0.05 vs. IESS group, %*P* < 0.05 vs. IESS+ACTH group, @*P* < 0.05 vs. IESS+anti-HMGB1 group) (one-way ANOVA).

The ELISA detection results (Figure 4) showed that the expressions of IL-1β, IL-2R, IL-8 and TNF-α in the IESS+NS group and IESS group were significantly higher than those in the BC group and NC group; compared with the IESS+NS group and IESS group, the expressions of IL-1β, IL-2R, IL-8 and TNF-α in the IESS+ACTH group, IESS+anti-HMGB1 group and IESS+ACTH+anti-HMGB1 group were significantly decreased. These results indicated that ACTH, anti-HMGB1 and their combination inhibited the expressions of IL-1β, IL-2R, IL-8 and TNF-α in the brain tissues of IESS young rats, with the combination intervention exerting the most significant effect.

### 3.5 Immunohistochemical detection results

**Figure 5:**
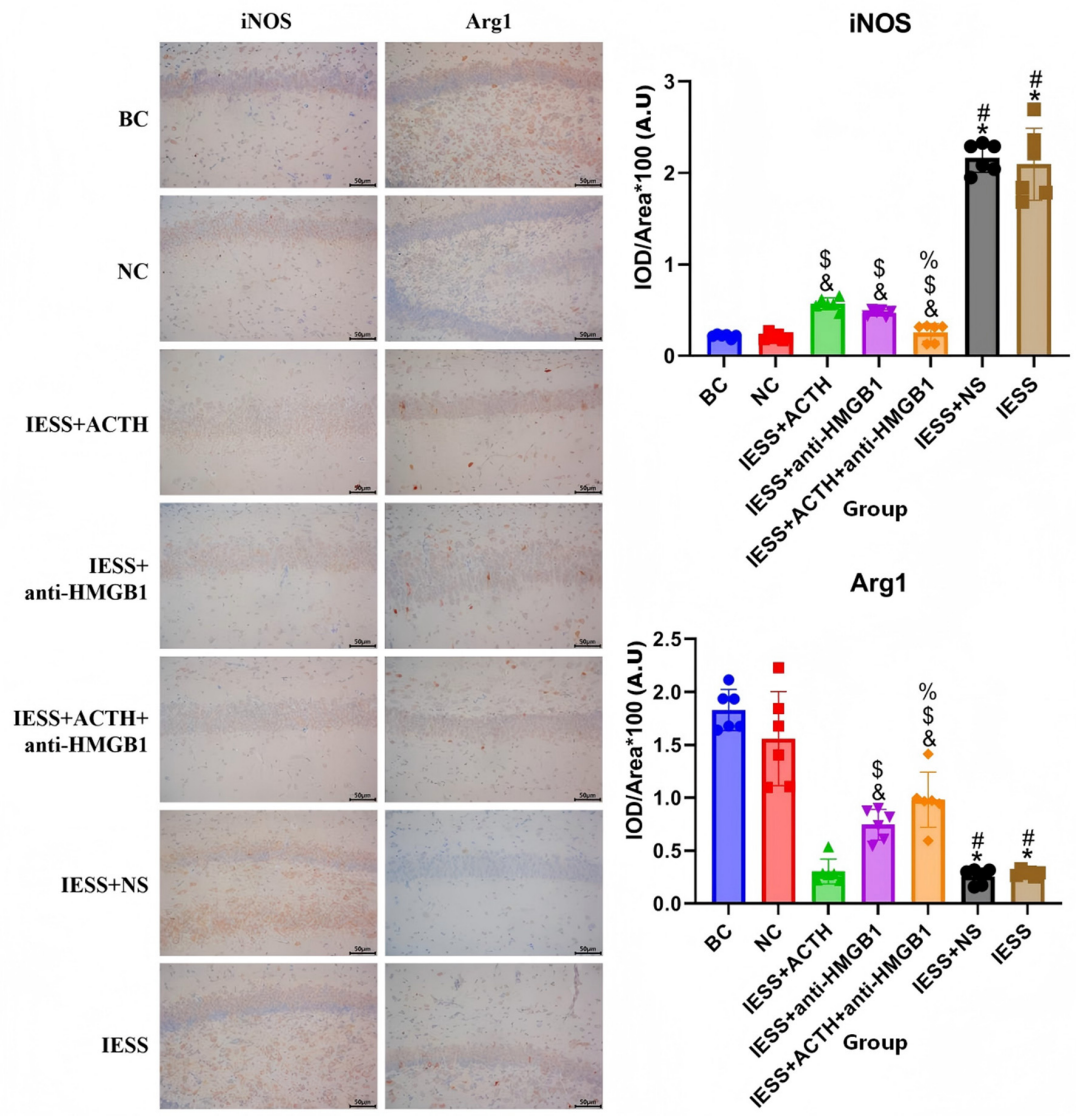
Immunohistochemical detection results. Experimental grouping: Non-model group (pups from non-prenatal stress pregnant rats) was divided into blank control (BC, no treatment) and negative control (NC, normal saline instead of drugs); Model group (pups from prenatal stress pregnant rats, IESS model induced by prenatal stress + NMDA intraperitoneal injection) was divided into 5 subgroups: Model I (IESS+ACTH, 100 IU/kg intraperitoneal injection), Model II (IESS+anti-HMGB1, 300 μg/kg intraperitoneal injection), Model III (IESS+ACTH+anti-HMGB1), Model IV (IESS+NS, equal volume normal saline), Model V (IESS, positive control, no treatment); Detection indicator: the expression of inducible nitric oxide synthase (iNOS) and arginase 1 (Arg1) in the brain tissues (hippocampus). Detection method: Immunohistochemical detection; Statistical significance description: (*P < 0.05 vs. BC group, #*P* < 0.05 vs. NC group, &*P* < 0.05 vs. IESS+NS group, $*P* < 0.05 vs. IESS group, %*P* < 0.05 vs. IESS+ACTH group) (one-way ANOVA).

The immunohistochemical detection results (Figure 5) showed that the expression of inducible nitric oxide synthase (iNOS) in the IESS+NS group and IESS group was significantly higher than that in the BC and NC groups; compared with the IESS+NS group and IESS group, the expression of iNOS was significantly decreased after intervention with ACTH, anti-HMGB1 and their combination, with the combination intervention showing the best effect. The expression of and arginase 1 (Arg1) in the brain tissues of the IESS+NS group and IESS group was significantly lower than that in the BC and NC groups; compared with the IESS+NS group and IESS group, ACTH intervention showed an increasing trend in Arg1 expression with no statistical significance (*P*>0.05), while anti-HMGB1 and the combination of ACTH and anti-HMGB1 significantly increased Arg1 expression, with the combination intervention exerting the best effect. These results indicated that ACTH, anti-HMGB1 and their combination could inhibit inflammation in the epileptic model and increase the expression of anti-inflammatory factors.

### 3.6 Double immunofluorescence staining detection results

**Figure 6:**
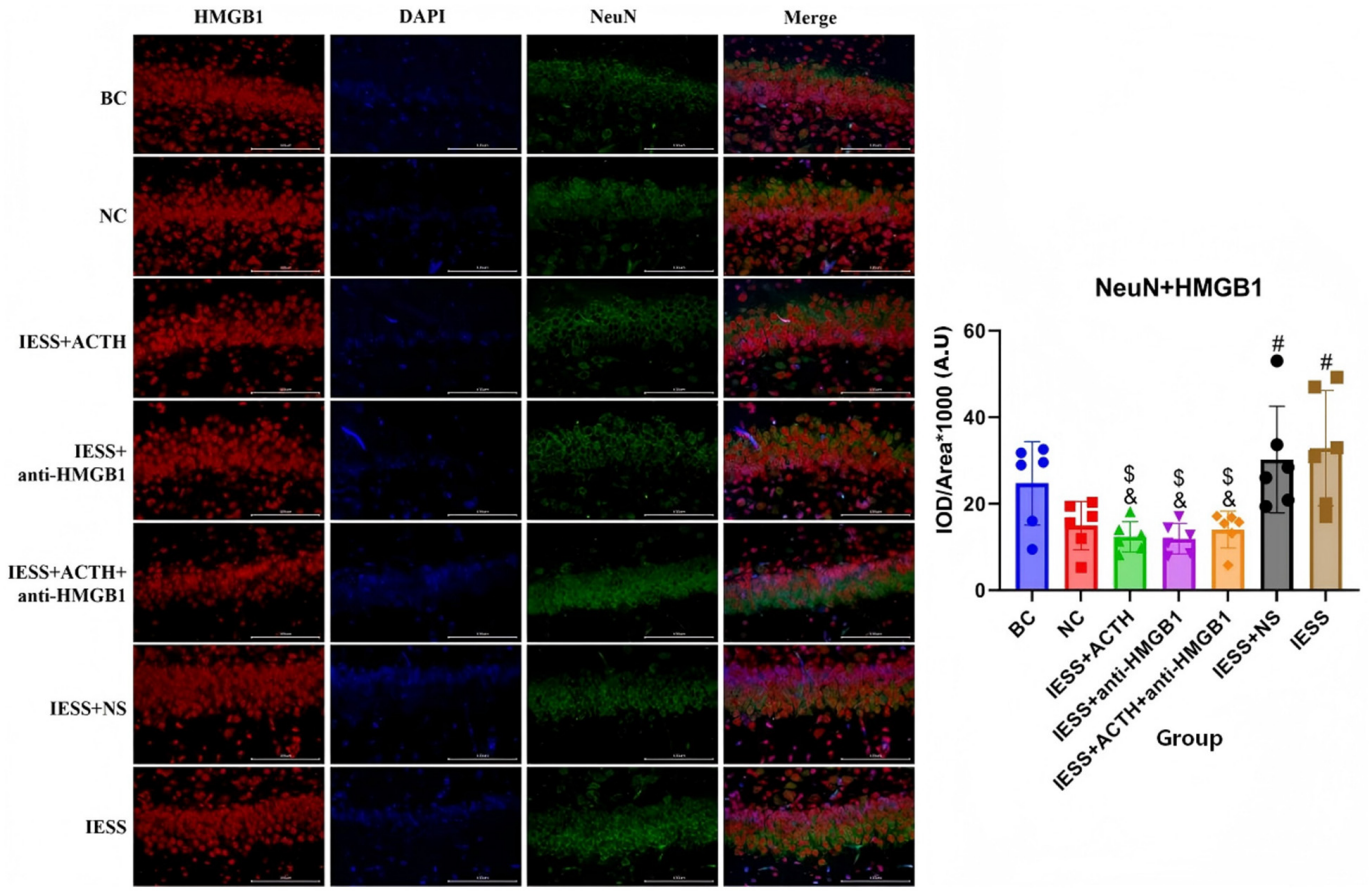
Double immunofluorescence staining results of NeuN and HMGB1. Experimental grouping: Non-model group (pups from non-prenatal stress pregnant rats) was divided into blank control (BC, no treatment) and negative control (NC, normal saline instead of drugs); Model group (pups from prenatal stress pregnant rats, IESS model induced by prenatal stress + NMDA intraperitoneal injection) was divided into 5 subgroups: Model I (IESS+ACTH, 100 IU/kg intraperitoneal injection), Model II (IESS+anti-HMGB1, 300 μg/kg intraperitoneal injection), Model III (IESS+ACTH+anti-HMGB1), Model IV (IESS+NS, equal volume normal saline), Model V (IESS, positive control, no treatment); Detection indicator: the colocalization expression of NeuN+HMGB1 in the hippocampal tissues. Detection method: Double immunofluorescence staining; Statistical significance description: (*P < 0.05 vs. BC group, #*P* < 0.05 vs. NC group, &*P* < 0.05 vs. IESS+NS group, $*P* < 0.05 vs. IESS group) (one-way ANOVA).

**Figure 7:**
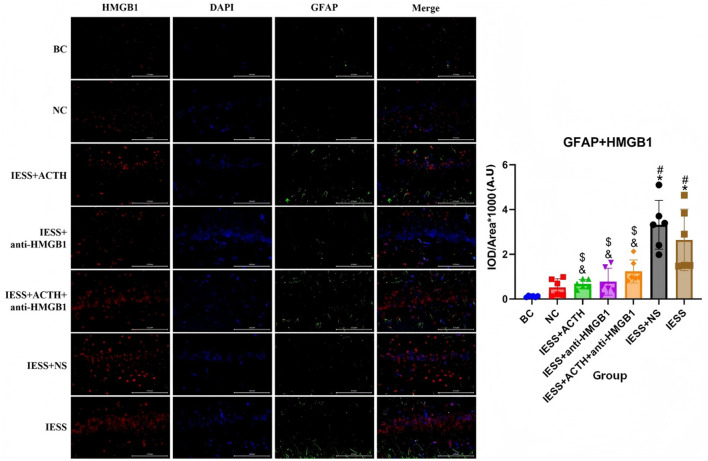
Double immunofluorescence staining results of GFAP and HMGB1. Experimental grouping: Non-model group (pups from non-prenatal stress pregnant rats) was divided into blank control (BC, no treatment) and negative control (NC, normal saline instead of drugs); Model group (pups from prenatal stress pregnant rats, IESS model induced by prenatal stress + NMDA intraperitoneal injection) was divided into 5 subgroups: Model I (IESS+ACTH, 100 IU/kg intraperitoneal injection), Model II (IESS+anti-HMGB1, 300 μg/kg intraperitoneal injection), Model III (IESS+ACTH+anti-HMGB1), Model IV (IESS+NS, equal volume normal saline), Model V (IESS, positive control, no treatment); Detection indicator: colocalization expression of GFAP+HMGB1. Detection method: Double immunofluorescence staining; Statistical significance description: (*P < 0.05 vs. BC group, #*P* < 0.05 vs. NC group, &*P* < 0.05 vs. IESS+NS group, $*P* < 0.05 vs. IESS group) (one-way ANOVA).

**Figure 8:**
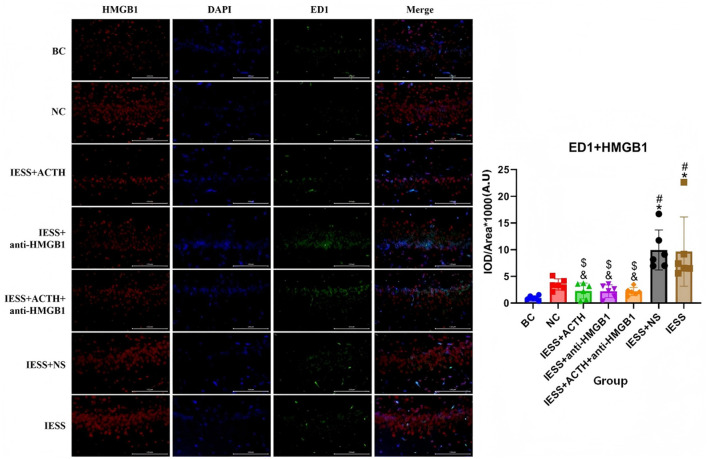
Double immunofluorescence staining results of ED1 and HMGB1. Experimental grouping: Non-model group (pups from non-prenatal stress pregnant rats) was divided into blank control (BC, no treatment) and negative control (NC, normal saline instead of drugs); Model group (pups from prenatal stress pregnant rats, IESS model induced by prenatal stress + NMDA intraperitoneal injection) was divided into 5 subgroups: Model I (IESS+ACTH, 100 IU/kg intraperitoneal injection), Model II (IESS+anti-HMGB1, 300 μg/kg intraperitoneal injection), Model III (IESS+ACTH+anti-HMGB1), Model IV (IESS+NS, equal volume normal saline), Model V (IESS, positive control, no treatment); Detection indicator: the colocalization expression of ED1+HMGB1. Detection method**:** Double immunofluorescence staining; Statistical significance description**:** (*P < 0.05 vs. BC group, #*P* < 0.05 vs. NC group, &*P* < 0.05 vs. IESS+NS group, $*P* < 0.05 vs. IESS group) (one-way ANOVA).

The results of NeuN+HMGB1 double immunofluorescence staining (Figure 6) showed that the colocalization expression of NeuN+HMGB1 in the hippocampal tissues of the IESS+NS group and IESS group was significantly higher than that in the NC group; compared with the IESS+NS group and IESS group, the colocalization expression of NeuN+HMGB1 was significantly decreased after intervention with ACTH, anti-HMGB1 and their combination. The results of GFAP+HMGB1 double immunofluorescence staining (Figure 7) showed that the colocalization expression of GFAP+HMGB1 in the IESS+NS group and IESS group was significantly higher than that in the BC group and NC group; compared with the IESS+NS group and IESS group, the colocalization expression of GFAP+HMGB1 was significantly decreased after treatment with ACTH, anti-HMGB1 and their combination. The results of ED1+HMGB1 double immunofluorescence staining (Figure 8) showed that the colocalization expression of ED1+HMGB1 in the IESS+NS group and IESS group was significantly higher than that in the BC group and NC group; compared with the IESS group and IESS+NS group, the colocalization expression of ED1+HMGB1 was significantly decreased after treatment with ACTH, anti-HMGB1 and their combination. These results indicated that ACTH, anti-HMGB1 and their combination could significantly inhibit the expression of HMGB1 in neurons, astrocytes and activated microglia in the IESS model.

## 4 Discussion

IESS is a severe age-dependent epileptic encephalopathy predominantly affecting infancy, characterized by epileptic spasms, hypsarrhythmia on electroencephalogram and developmental delay, with a poor prognosis and unclear pathogenesis. ACTH, as one of the most classic first-line drugs for clinical treatment of IESS (40–42), has been widely confirmed for its efficacy, but its mechanism of action remains not fully elucidated, and a considerable number of patients have poor efficacy, drug resistance or high recurrence rate (43, 44). Therefore, it is crucial to study the pathogenesis of IESS from a new perspective and explore new therapeutic targets. In recent years, numerous studies have confirmed that neuroinflammation plays a key role in the pathogenesis of epilepsy, among which the HMGB1-TLR4 signaling pathway is the core mediator of neuroinflammatory responses in epilepsy (45, 46). However, its role in IESS remains unclear. This study established an IESS rat model induced by prenatal stress (PS) combined with N-methyl-D-aspartate (NMDA) to systematically investigate the role of HMGB1-TLR4-mediated neuroinflammatory mechanism in IESS, and evaluate the therapeutic effects of ACTH, HMGB1 neutralizing antibody (anti-HMGB1) and their combination on IESS rats and their effects on neuroinflammation, aiming to provide new theoretical basis and experimental support for the study of pathogenesis and targeted therapy of IESS.

### 4.1 Prenatal stress combined with NMDA can stably establish the IESS model in young rats

Establishing a stable and clinically relevant animal model is the basis for investigating the pathogenesis of IESS and evaluating therapeutic strategies. Previous studies have shown that prenatal stress can increase the sensitivity of young rats to NMDA-induced spasms and induce increased neuronal excitability, which is consistent with the developmental pathogenesis of IESS (47, 48). As a glutamate receptor agonist, NMDA can induce epileptic spasms in the developmental window of rodents corresponding to human infancy, and its combined application with prenatal pretreatment (such as betamethasone or prenatal stress) has been widely used to establish animal models of IESS (49). In this study, pregnant rats in the PS group were subjected to 5 min of 4 °C cold water immersion plus 10 min of warm air drying daily from gestational day 1 to simulate prenatal stress, and their pups were intraperitoneally injected with 7.5 mg/kg NMDA on postnatal day 12 (P12) to induce infantile spasm-like seizures. All model rats included in this study exhibited curling-up spasm and involuntary choreiform movements of limbs after tonic-clonic seizures, indicating the successful establishment of the IESS model in young rats, which was consistent with the conclusion of previous studies that “prenatal stress combined with NMDA can successfully induce spasms” (49). Notably, compared with single NMDA induction, the model induced by PS combined with NMDA is more in line with the clinical pathogenesis of IESS—clinical studies have also found that children with IESS are often associated with adverse prenatal factors (such as maternal stress) (50). Therefore, the IESS model established by prenatal stress combined with NMDA provides a more reliable experimental platform for subsequent mechanism research and therapeutic effect evaluation of IESS.

### 4.2 Activation of the HMGB1-TLR4 signaling pathway in the brain tissues of IESS young rats mediates neuroinflammatory responses and participates in the pathogenesis of IESS

The core finding of this study is that the HMGB1-TLR4 signaling pathway is significantly activated in the brain tissues of IESS rats, and the activation of this pathway mediates neuroinflammatory responses and participates in the pathogenesis of IESS. As a non-histone nuclear protein, HMGB1 is involved in DNA transcription, translation and repair under physiological conditions; under pathological conditions such as epilepsy, it can be released from the nucleus to the cytoplasm and extracellular space, and acts as a pro-inflammatory cytokine to bind to downstream receptors such as TLR4, thereby activating neuroinflammatory responses. As a key pattern recognition receptor in the innate immune system, TLR4 can trigger the activation of downstream signaling pathways such as NF-κB after binding to HMGB1, leading to excessive release of pro-inflammatory factors (25–28) and aggravating neuronal damage and epileptic seizures. The qPCR and Western blot results of this study showed that the mRNA and protein expression levels of HMGB1 and TLR4 in the brain tissues of IESS rats were significantly higher than those in the control group, which was consistent with the conclusion of previous studies that “HMGB1 and TLR4 are highly expressed in the epileptic tissues of animal models and human epilepsy patients” (29). Multiple previous studies have shown that the hippocampal tissue is one of the important central structures involved in the pathogenesis of IESS (51, 52), and the hippocampus plays an important role in the regulation of neuroinflammation in epilepsy (53, 54). Therefore, this study further explored the correlation between the types of neural cells in the hippocampal tissue and HMGB1. The double immunofluorescence staining results showed that HMGB1 was highly expressed in neurons (NeuN-positive), astrocytes (GFAP-positive) and activated microglia (ED1-positive) in the hippocampal tissues of IESS rats, suggesting that these three types of cells are important sources of HMGB1 release and jointly participate in the HMGB1-TLR4-mediated neuroinflammatory response in IESS. This is consistent with the view that “neuroinflammation in epilepsy is a comprehensive response involving neurons and neuroglial cells, which interact to form a vicious circle between inflammation and epileptic seizures” (55).

iNOS and Arg1 are classic markers of pro-inflammatory and anti-inflammatory immune responses, often used as important reference indicators for M1-type pro-inflammatory and M2-type anti-inflammatory macrophage/microglial phenotypes, respectively. The results of this study showed that the expression of Arg1 was significantly decreased and the expression of iNOS was significantly increased in the brain tissues of IESS rats, indicating an overall shift to pro-inflammatory immune responses in the brain tissues and an imbalance in the expression of neuroinflammation-related molecules, which may be involved in the occurrence and development of neuroinflammation. Consistently, the ELISA results showed that the expressions of pro-inflammatory cytokines IL-1β, IL-2R, IL-8 and TNF-α were significantly increased in the brain tissues of IESS rats. These pro-inflammatory cytokines can directly or indirectly affect neuronal electrical activity, enhance neuronal excitability, damage the blood-brain barrier, further promote the occurrence and development of epileptic seizures, and form a vicious circle between neuroinflammation and epilepsy (55). Notably, this study showed that the elevation of these pro-inflammatory cytokines in IESS rats was closely related to the activation of the HMGB1-TLR4 pathway, suggesting that HMGB1-TLR4 pathway-mediated neuroinflammation in IESS rats is characterized by an imbalance between pro-inflammatory and anti-inflammatory factors, and this imbalance further aggravates brain damage in IESS.

### 4.3 ACTH, Anti-HMGB1 alone and in combination can improve epileptic seizures in IESS rats by inhibiting the HMGB1-TLR4 pathway and alleviating neuroinflammation

This study further evaluated the therapeutic effects of ACTH, anti-HMGB1 and their combination on IESS rats, explored their mechanisms of action, and sought new targets for clinical treatment. As mentioned above, the HMGB1-TLR4 signaling pathway is significantly activated in the brain tissues of IESS rats, and the activation of this pathway mediates neuroinflammatory responses and participates in the pathogenesis of IESS. Therefore, this study used a specific neutralizing antibody against HMGB1 (anti-HMGB1) for intervention to block the binding of HMGB1 to TLR4, thereby inhibiting the activation of the HMGB1-TLR4 pathway and neuroinflammatory responses, and compared it with ACTH. The results of this study showed that ACTH, anti-HMGB1 alone and in combination significantly prolonged the seizure latency, reduced the seizure severity score, and improved the pathological changes of neuroinflammation in IESS young rats, with the combination intervention showing the optimal effect. At the mechanistic level, all three intervention methods significantly down-regulated the expressions of HMGB1 and TLR4 in the brain tissues of IESS rats, inhibited the expression of HMGB1 in neurons, astrocytes and activated microglia, decreased the expressions of iNOS and pro-inflammatory factors such as IL-1β, IL-2R, IL-8 and TNF-α, and increased the expression of the anti-inflammatory factor Arg1. This suggests that both ACTH and anti-HMGB1 exert therapeutic effects on IESS by inhibiting the HMGB1-TLR4-mediated neuroinflammatory pathway, and their combination can produce a synergistic effect, which may be related to the complementary regulatory effects of the two intervention methods on the HMGB1-TLR4 pathway and neuroinflammation.

The mechanism of action of ACTH in the treatment of IESS has long been a research hotspot. Previous studies have shown that ACTH can reduce the expression of corticotropin-releasing hormone (CRH) in the limbic system through steroid-dependent and steroid-independent pathways, thereby reducing neuronal excitability and reducing epileptic seizures (56). This study found that ACTH can significantly inhibit the activation of the HMGB1-TLR4 pathway and the release of pro-inflammatory factors, and reduce the seizure severity score and improve the pathological changes of neuroinflammation, which provides a new perspective for explaining the mechanism of action of ACTH in the treatment of IESS, indicating that ACTH can control IESS by regulating the expression and release of HMGB1, thereby inhibiting the HMGB1-TLR4-mediated neuroinflammatory response. For anti-HMGB1, the results of this study further confirmed its therapeutic potential in IESS, which was consistent with the conclusions of multiple previous studies that “anti-HMGB1 can reduce the incidence and severity of epileptic seizures by blocking the HMGB1-TLR4 pathway” (57–60). More importantly, the therapeutic effect of the combination of ACTH and anti-HMGB1 was superior to single intervention, suggesting that the combination of traditional hormonal therapy and targeted anti-inflammatory therapy may become a new strategy for the treatment of IESS, especially suitable for patients with poor response to single ACTH treatment.

### 4.4 Strengths and limitations of this study

This study has the following strengths: First, a stable IESS rat model was established by PS combined with NMDA induction, which is close to the developmental and clinical pathogenesis of IESS, providing a reliable experimental basis for subsequent studies; Second, the role of HMGB1-TLR4-mediated neuroinflammatory mechanism in IESS was systematically investigated from multiple levels including mRNA, protein, cellular localization and cytokine levels, and the therapeutic effects of ACTH, anti-HMGB1 and their combination were verified, enriching the understanding of the pathogenesis and therapeutic targets of IESS; Third, the synergistic therapeutic effect of the combination of ACTH and anti-HMGB1 was found, providing a new idea for the clinical treatment of IESS. However, it is important to note that long-term and continuous inhibition of HMGB1 activity is not entirely safe. Previous studies have demonstrated that prolonged blockade of the HMGB1 signaling pathway can impair the body's ability to regulate peripheral immune homeostasis, disrupt the balance of immune suppression, and thereby induce or exacerbate inflammatory injury in peripheral organs (61). Therefore, if anti-HMGB1 antibodies are to be developed for the clinical treatment of IESS in the future, strict control of the treatment course and dosage will be required to balance the benefits of central anti-inflammatory and anti-epileptic effects against the potential risks of peripheral immune dysregulation.

However, this study also has some limitations that need to be further improved in subsequent studies: First, this study was based on a rat model, and the results need to be further verified in clinical samples of patients with IESS to clarify the clinical correlation of the HMGB1-TLR4 pathway and the therapeutic potential of combined intervention; Second, this study only evaluated the short-term therapeutic effects of the interventions, and their long-term effects on neural development and epileptogenesis still need to be further explored; Third, the specific and in-depth molecular mechanism by which HMGB1-TLR4 pathway-mediated neuroinflammation participates in the pathogenesis of IESS is not clear, and subsequent studies need to focus on the upstream regulatory factors of this pathway to further elucidate its mechanism of action; Fourth, this study did not explore the optimal dosage and course of administration of ACTH and anti-HMGB1, and subsequent experiments are needed to optimize the administration regimen to provide more precise guidance for clinical application.

## 5 Conclusion

In summary, this study confirmed that prenatal stress combined with NMDA can stably establish the IESS model in young rats, in which the HMGB1-TLR4 signaling pathway in the brain is activated, thereby mediating neuroinflammatory responses and participating in the pathogenesis of IESS. ACTH, anti-HMGB1 alone and in combination can alleviate neuroinflammation by inhibiting this pathway to improve epileptic seizures in IESS rats, among which the combination intervention exerts the best effect. The results of this study not only deepen the understanding of the pathogenesis of IESS but also provide a new theoretical basis and potential therapeutic strategy for the clinical treatment of IESS. Subsequent studies should focus on verifying the clinical significance of the HMGB1-TLR4 pathway, exploring the long-term therapeutic effects of combined intervention, and promoting the translation of experimental results into clinical practice

## Data Availability

The original contributions presented in the study are included in the article, further inquiries can be directed to the corresponding author. Requests to access the datasets should be directed to 1341220182@qq.com.
